# Research priorities for adolescent health in low- and middle-income countries: A mixed-methods synthesis of two separate exercises

**DOI:** 10.7189/jogh.08.010501

**Published:** 2018-06

**Authors:** Jason M Nagata, Sejal Hathi, B Jane Ferguson, Michele J Hindin, Sachiyo Yoshida, David A Ross

**Affiliations:** 1 Division of Adolescent and Young Adult Medicine, Department of Pediatrics, University of California, San Francisco, San Francisco, California, USA; 2 Department of Pediatrics, Stanford University, Palo Alto, California, USA; 3 School of Medicine, Stanford University, Palo Alto, California, USA; 4 Healthy Adolescents & Young Adults Research Unit, Africa Health Research Institute, Mtubatuba, South Africa; 5 London School of Hygiene & Tropical Medicine, London, United Kingdom; 6 Department of Population, Family and Reproductive Health, Johns Hopkins Bloomberg School of Public Health, Baltimore, Maryland, USA; 7 The Population Council, New York, New York, USA; 8 Department of Maternal, Newborn, Child, and Adolescent Health, World Health Organization, Geneva, Switzerland

## Abstract

**Background:**

In order to clarify priorities and stimulate research in adolescent health in low- and middle-income countries (LMICs), the World Health Organization (WHO) conducted two priority-setting exercises based on the Child Health and Nutrition Research Initiative (CHNRI) methodology related to 1) adolescent sexual and reproductive health and 2) eight areas of adolescent health including communicable diseases prevention and management, injuries and violence, mental health, non-communicable diseases management, nutrition, physical activity, substance use, and health policy. Although the CHNRI methodology has been utilized in over 50 separate research priority setting exercises, none have qualitatively synthesized the ultimate findings across studies. The purpose of this study was to conduct a mixed-method synthesis of two research priority-setting exercises for adolescent health in LMICs based on the CHNRI methodology and to situate the priority questions within the current global health agenda.

**Methods:**

All of the 116 top-ranked questions presented in each exercise were analyzed by two independent reviewers. Word clouds were generated based on keywords from the top-ranked questions. Questions were coded and content analysis was conducted based on type of delivery platform, vulnerable populations, and the Survive, Thrive, and Transform framework from the United Nations Global Strategy for Women’s, Children’s, and Adolescents’ Health, 2016-2030.

**Findings:**

Within the 53 top-ranked intervention-related questions that specified a delivery platform, the platforms specified were schools (n = 17), primary care (n = 12), community (n = 11), parenting (n = 6), virtual media (n = 5), and peers (n = 2). Twenty questions specifically focused on vulnerable adolescents, including those living with HIV, tuberculosis, mental illness, or neurodevelopmental disorders; victims of gender-based violence; refugees; young persons who inject drugs; sex workers; slum dwellers; out-of-school youth; and youth in armed conflict. A majority of the top-ranked questions (108/116) aligned with one or a combination of the Survive (n = 39), Thrive (n = 67), and Transform (n = 28) agendas.

**Conclusions:**

This study advances the CHNRI methodology by conducting the first mixed-methods synthesis of multiple research priority-setting exercises by analyzing keywords (using word clouds) and themes (using content analysis).

Approximately 1.2 billion people, almost one sixth of the world’s population, are adolescents [1]. Nearly 90 percent of adolescents live in low- and middle-income countries (LMICs) [1]. As the era of the Millennium Development Goals fades, young people are critical to the post-2015 development agenda. Young people will be key beneficiaries—and must be key partners—of the global Sustainable Development Goals. For that reason, concern for their health has surged to the forefront of the global health agenda, a trend exemplified by the specific reference to adolescent health in the United Nations Global Strategy for Women’s, Children’s, and Adolescents’ Health, 2016-2030 [2]. This global strategy stresses that adolescents must not only survive, but also thrive in order to transform the world. This is the first time a global strategy has specifically highlighted the needs of adolescents.

In order to stimulate research in adolescent health in LMICs, the World Health Organization (WHO) used the Child Health and Nutrition Research Initiative (CHNRI) methodology for two separate research priority-setting exercises on 1) seven areas of adolescent sexual and reproductive health including maternal health; contraception; gender-based violence; HIV treatment and care; abortion; family planning/reproductive health and HIV service integration; and sexually transmitted infections and human papillomavirus infection [3]; and 2) eight other areas of adolescent health including communicable diseases prevention and management; injuries and violence; mental health; non-communicable diseases management; nutrition; physical activity; substance use; and health policy [4].

At a WHO technical consultation on research priorities for adolescent health in October 2015 [5], experts discussed combining the results from the two exercises. The consensus was that it would not be valid to quantitatively rank between all adolescent health research areas because different participants and scoring criteria had been used in the two exercises. However, a recommendation emerged to undertake additional qualitative analysis across platforms and common themes within the different health areas [5]. This “horizontal” analysis would bridge the 15 “vertical” health areas covered in the two adolescent research priority-setting exercises. The aim of this qualitative synthesis, defined as an intentional and coherent approach to analyzing data across qualitative studies [6], was to enable a more holistic assessment of adolescent health research priorities. It might also better inform countries’ adolescent health policies and programming, much of which is structured according to delivery platforms or settings (eg, schools or primary care). Synthesizing by platform also emphasizes intervention research (including development/testing and implementation/delivery research), which is specifically tailored toward translating research evidence into practice, policies, and programming in the real world [7]. Finally, this horizontal analysis might clarify key themes or patterns within the top research questions for funding agencies.

Although the CHNRI method has become the most common methodology for identifying global research priorities and has been utilized in over 50 research priority-setting exercises [8-10], none have qualitatively synthesized the ultimate findings. One CHNRI exercise on maternal and perinatal health [11] integrated questions identified in a previous newborn health exercise [12], but this synthesis occurred prior to scoring. Another study extracted the highest-ranked intervention-related questions from previous CHNRI exercises, and conducted yet another CHNRI exercise on top of these, to identify priorities for implementation research in global maternal, newborn, child, and adolescent health in Canada [13]. However, neither of these exercises, nor any others to our knowledge, have analyzed the outcomes of more than one priority-setting exercise (ie, the top-ranked research questions) after scoring and ranking using qualitative research methods. The two CHNRI exercises synthesized in this article [3,4] are the only two completed to date on adolescent health research and were designed to span most of the major areas of adolescent health. Given their combined comprehensiveness across different areas of adolescent health research, these two exercises would be ideal for synthesis.

The objective of this study was to synthesize findings from two prior adolescent health research priorities exercises in LMICs, particularly across delivery platforms and themes, as well as to assess how the top-ranked research questions related to the adolescent health priorities listed in the United Nations Global Strategy for Women’s, Children’s and Adolescents’ Health [2]. These objectives were identified during the WHO technical consultation on research priorities for adolescent health, which involved over 30 global experts in the field of adolescent health [5].

## METHODS

The methods used in the two priority-setting exercises on adolescent health research were based on the CHNRI methodology for prioritization of health research [8,14-18]. The CHNRI methodology have been described in detail, including identification of participants, generation of research questions, and scoring protocols [3,4]. The five criteria for ranking the research questions were clarity, answerability, impact, implementation, and equity. These criteria were weighted equally in the adolescent sexual and reproductive health priority-setting exercise [3] whereas they were weighted based on published guidelines from CHNRI stakeholders [15] in the priority-setting exercise for the eight other areas of adolescent health [4]. These adolescent research priority-setting exercises identified 116 top-ranked research questions (36 in the seven domains of adolescent sexual and reproductive health [3], and 80 in the eight other areas of adolescent health [4]). All top-ranked questions were included because they had been identified as important to the field of adolescent health by participants. In this mixed-methods synthesis, we analyzed these 116 research questions at the individual word level using word cloud analysis and at the thematic level using content analysis.

### Word cloud analysis

Word clouds are visual representations of text that depict the words that occur most often within the text [19]. They are constructed by positively correlating the font size of depicted words with the word frequency. In this study, the text of all 116 top-ranked questions [1,2] was standardized to American English (eg, “behavior”), recoded to avoid duplication (eg, combining “intervention” and “interventions”, and non-specific words (eg, “a,” “the,” “and”) were removed. In addition, we excluded the words “adolescent,” “adolescents,” “adolescence,” and “health” from analysis because all questions were by definition about adolescents’ health. The resulting text was uploaded to the online word cloud generator, Word Art [20].

### Content analysis

Content analysis was performed by two independent reviewers (JMN and SH) to identify major themes across the two adolescent health research priority-setting exercises [21,22]. The questions were analyzed and coded based on three objectives for the mixed-methods synthesis: 1) identification of key platforms or settings used to deliver adolescent health interventions, 2) the frequency with which specific vulnerable groups and genders of adolescents featured among priority questions, and 3) alignment with the Survive, Thrive, and Transform framework from the United Nations Global Strategy for Women’s, Children’s, and Adolescents’ Health.

All top research questions (n = 116) were coded using the three criteria described above (intervention delivery platforms; vulnerable populations; and Survive, Thrive, and Transform). Questions that were relevant to the above criteria were then grouped together. Content analysis was performed on these grouped questions and themes were identified by intervention delivery platforms (ie, primary care, community, schools, parenting, etc.); by adolescent sub-populations specifying vulnerability (eg, sex workers, refugees, out of school youth); and by gender. In accordance with the United Nations Global Strategy for Women’s, Children’s, and Adolescents’ Health [2], questions specifically related to Survive, Thrive, and Transform were also identified, and a Venn diagram was created to highlight areas of difference and overlap between these three domains. For each domain and its areas of overlap, content analysis was used to synthesize relevant questions into key themes.

## RESULTS

**Figure 1:**
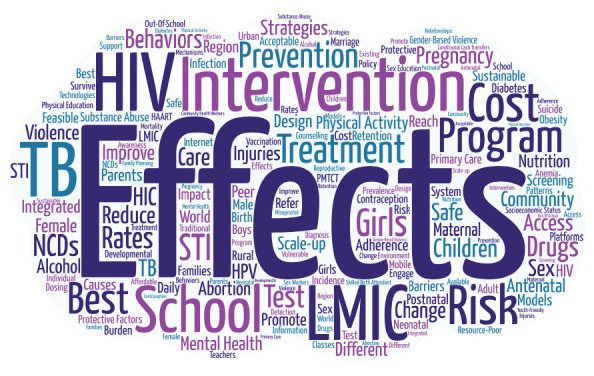
Word cloud of top-ranked questions from two adolescent research priority-setting exercises.

Based on the word cloud analysis (**Figure 1**), the most commonly repeated words were “effects” (n = 32 mentions), “intervention” (n = 25), “school” (n = 18), “LMIC” (n = 17), “program” (n = 14), “HIV” (n = 14), “treatment” (n = 13), “risk” (n = 13), “TB” (n = 11), “prevention” (n = 10), “cost” (n = 10), “physical activity” (n = 10), “pregnancy” (n = 10), “best” (n = 9), “behaviors” (n = 9), “community” (n = 9), “strategies” (n = 9), and “mental health” (n = 9). “Gender” was mentioned on six occasions and “sex” on four occasions. “Boys” received two mentions to “girls”’ eight. Words related to maternal care for adolescents, such as “birth,” “PMTCT (prevention of mother-to-child transmission of HIV),” and “skilled birth attendants,” received 2 mentions each.

Six intervention delivery platforms were specified in the top-ranked questions: schools (n = 17), primary care (n = 12), community (n = 11), parenting (n = 6), virtual media (n = 5), and peers (n = 2) (**Table 1**). The specific questions for each intervention delivery platform can be found in Table S1 in **Online Supplementary Document(Online Supplementary Document)**. School-based interventions included instruction in skills such as swimming and water survival, sexual education, and prevention activities related to sexually transmitted infections (STI), gender-based violence, mental health, obesity and physical activity. The importance of education as a determinant of adolescent health was also underscored by top-ranked questions that related to reaching out-of-school adolescents and on decreasing school attrition with conditional cash transfer programs.

**Table 1:** Priority themes organized by delivery platform for adolescent health interventions

Schools **(n = 17)**
Incorporation of more safe routes to schools initiatives
Teaching swimming and water survival, gender-based violence, sexual education, STI prevention in schools
School-based interventions for mental health, obesity, physical activity (including 60 min of moderate to vigorous activity daily)
Identification of key interventions for school health provision
Conditional cash transfer programs to keep girls in schools
Strategies for reaching out-of-school adolescents
**Primary care** (n = 12)
Integration of primary care health services with: mental health, reproductive health (antenatal, postnatal), community health
Identification and implementation of evidence-based screening and prevention interventions: alcohol use, mental health, adolescents with intellectual disabilities and neurodevelopmental delays
Adolescent-friendly health services: confidentiality, anonymity, social services offered, counseling services offered, speed of results
Barriers for adolescents to accessing services
**Community** (n = 11)
Community health workers: accessibility and acceptability to adolescents
Training of community health workers on: adolescent sexual and reproductive health, gender-based violence
Understanding community risk factors for: injuries, violence, drowning, communicable diseases
Community-based programs: obesity, physical activity, substance use, gender-based violence, STI counseling and testing, HPV vaccination
**Parenting** (n = 6)
Parenting programs for: the prevention of mental health disorders, management of substance use disorders
Programs for parents to create physical activity-friendly environments
**Virtual media (ie, internet, mobile phones, electronics, social media)** (n = 5)
Identification of communication strategies that work best for adolescents
Identification of how adolescents use information technologies
Using cell phones, the internet, other technologies to effectively provide information, referrals, and treatment for adolescents
Understanding virtual means to promote healthy choices
**Peer education** (n = 2)
Impact of peer education on substance abuse
Peer education for improving retention in care for adolescents with HIV and/or tuberculosis

HIV – human immunodeficiency virus; HPV – human papillomavirus; STI – sexually transmitted infection

Related to the community delivery platform or setting, two questions explored the potential of community health workers, asking 1) how acceptable and accessible they are to adolescents and 2) how they might be more effectively trained on adolescent-specific gender-based violence and sexual and reproductive health issues. Other questions raised the possibility of interventions on a range of health issues – obesity prevention, physical activity, substance use, gender-based violence, sexually transmitted infection counseling and testing, and human papilloma virus vaccination – that might be delivered in community settings. Finally, several questions sought to better characterize community-level risk factors for injuries, violence, drowning, and communicable diseases.

Related to the primary care platform or setting, several questions addressed the integration of health services, including physical, mental, and reproductive (including antenatal and postnatal) health services. Another theme coalesced around the identification of evidence-based screening and prevention interventions for alcohol abuse, mental health, intellectual disabilities, and neurodevelopmental disorders. Creation of adolescent-friendly health services was also a common theme, emphasizing confidentiality, anonymity, social services and counseling, and rapid results (ie, results of laboratory testing available during the same visit when possible, such as rapid HIV testing). Finally, a major theme concerned identifying barriers to adolescents accessing services for HIV, tuberculosis, and contraception.

The final three health platforms – parenting, virtual media, and peer education – appeared less frequently but nonetheless suggested important avenues for influencing adolescents. Questions explored the role of parents in addressing mental health, substance use, and physical inactivity at home. Questions related to virtual media suggested the need for investigation of how to best use virtual media to promote healthy choices among adolescents. Peer education was proposed as a strategy for addressing substance use and improving retention in HIV and tuberculosis care, among other conditions.

Across the top-ranked research questions, several vulnerable adolescent populations were identified (**Table 2**). The most frequently mentioned vulnerable populations included adolescents living with HIV (n = 15), tuberculosis (n = 9), and mental illness (n = 8). Questions about other vulnerable populations included victims of gender-based violence (n = 7), out-of-school adolescents (n = 6), and adolescents living in resource-poor communities (n = 3). Questions that related specifically to adolescents at risk of suicide or self-harm behaviors; adolescents living with neurodevelopmental disorders, developmental delays, and intellectual disabilities; sex workers; refugees; slum dwellers; youth in armed conflict; and young people who inject drugs also appeared among the top-ranked questions.

**Table 2:** Vulnerable adolescent populations identified in research priority-setting exercises by frequency

Adolescents living with HIV (n = 15)
Adolescents living with tuberculosis (n = 9)
Adolescents living with mental illness (n = 8)
Gender-based violence victims (n = 7)
Out-of-school adolescents (n = 6)
Adolescents living in resource-poor communities (n = 3)
Adolescents at risk for suicide (n = 2)
Adolescents living with neurodevelopmental disorders (n = 2)
Adolescents engaging in self-harm behaviors (n = 1)
Adolescents living with developmental delays (n = 1)
Adolescents living with intellectual disabilities (n = 1)
Adolescents who inject drugs (n = 1)
Refugees (n = 1)
Sex workers (n = 2)
Slum dwellers (n = 1)
Youth in armed conflict (n = 1)

**Figure 2:**
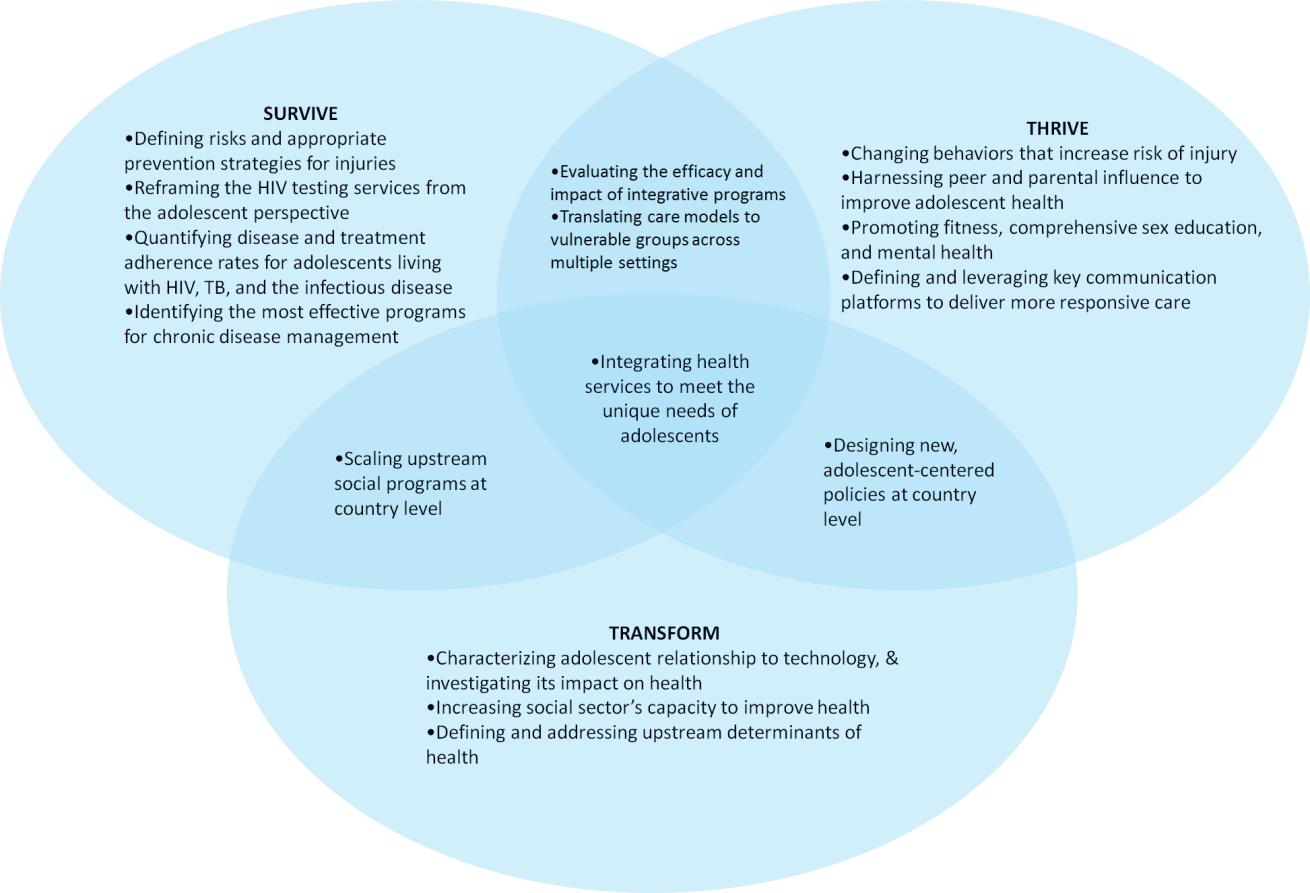
Venn diagram of adolescent health research priorities placed in the United Nations Global Strategy for Women’s, Children’s, and Adolescents’ Health “Survive, Thrive, and Transform” framework.

Of the top-ranked questions, 108 aligned with one or a combination of the Global Strategy’s Survive, Thrive or Transform agendas (**Figure 2**). Thirty nine related to Survive; 67 to Thrive; and 28 to Transform (Table S2 in **Online Supplementary Document(Online Supplementary Document)**). Those addressing the Survive agenda primarily concerned defining the most effective prevention, monitoring, and treatment strategies for communicable and non-communicable diseases. Those addressing the Thrive agenda chiefly focused on leveraging key communication channels – including peers, parents and virtual media – to promote healthy behavior. And those addressing the Transform agenda explored the impact of non-health sectors on adolescent health. Additionally, nine questions related to both the Survive and Thrive agendas; seven to both Survive and Transform; and seven to both Thrive and Transform. Questions addressing both Survive and Thrive were united by their focus on prevention, particularly in vulnerable populations, to promote wellness. Those addressing both Survive and Transform sought to identify more scalable touchpoints, from schools to primary care, for engaging adolescents in their own health. And those addressing both Thrive and Transform asked how existing health delivery platforms might be reimagined to appeal more effectively to adolescents. Three questions – seeking interventions that holistically addressed adolescent health needs – fulfilled all three agendas.

## DISCUSSION

We present the results of the first mixed-methods synthesis of multiple research priority-setting exercises based on the CHNRI methodology. We combined the top-ranked questions identified in two separate adolescent health research priority-setting exercises that comprehensively spanned multiple areas of adolescent health research in LMICs to identify key words and themes. Key areas of analysis included delivery platforms for adolescent health interventions; vulnerable adolescent populations; and alignment with the Survive, Thrive, and Transform framework of the United Nations Global Strategy for Women’s, Children’s and Adolescents’ Health.

In the word cloud analysis, the dominance of the words “effects” and “intervention” suggests that a specific type of research was prioritized, namely studies to evaluate the effectiveness of interventions. Also “schools” were frequently mentioned, suggesting an emphasis on education and on schools as a key intervention delivery platform.

Questions related to gender and sex featured prominently in the research priority-setting exercises. Several questions addressed the feasibility and impact of gender-based violence interventions in community settings and in schools. Overall, girls were mentioned four times as frequently as boys in the world cloud analysis. Questions related to adolescent girls focused on interventions to keep girls in school and to improve their antenatal, perinatal, and postnatal care during pregnancy. In addition, several questions addressed specific health issues in adolescent girls, including anemia, self-harm and suicide, and burn injuries. Only one question, related to modalities for delivering integrated HIV and family planning services, focused exclusively on boys. These questions reflect a corresponding emphasis on adolescent girls in the global health research agenda [23].

Analysis across multiple health areas and specialties allowed for the identification of themes that transcend vertical programming structures. In particular, integration of services across subspecialties and across delivery platforms was a major focus of the questions. For instance, primary care was frequently positioned as a delivery platform where mental health and sexual and reproductive health, including antenatal and postnatal care, could be addressed in integrated programs. Similarly, analysis revealed opportunities to investigate the effectiveness of collaborations between community health workers, teachers, and school health workers. Virtual media technologies were also suggested as a tool to investigate novel adolescent-focused interventions related to health education, behavior change, referral, and treatment.

Furthermore, schools emerged as an important platform to research health interventions for adolescents. To this end, top-ranked research questions addressed incentives such as conditional cash transfer programs to keep girls in schools. Other questions explored potential health topics for schools, including sexual health education, STI prevention, gender-based violence reduction, and water survival and safety. Specific interventions for research evaluation in schools included physical education classes to promote moderate to vigorous physical activity and swimming classes to prevent drowning.

Many questions were moreover united by an intent to address how best to reduce inequalities in global adolescent health. Reducing inequalities remains a major global health priority as evidenced as its inclusion as one of the United Nations’ core Sustainable Development Goals [24]. Furthermore, the Lancet Commission on Investing in Health identifies the elimination of health inequities within a generation as a means of achieving the most dramatic gains in global health by 2035, a phenomenon referred to as the “grand convergence” [25]. Critical to this ambition is addressing the health needs of the most vulnerable populations, many of which are identified through analysis of the research priorities questions. A top research priority should therefore be to assess equity of access to health interventions for these vulnerable populations.

Finally, the meta-synthesis allowed for analysis of adolescent health research through a life course approach. For instance, the current health condition of adolescents will affect their future adult health, in addition to the next generation. This life course model reflects the new Global Strategy, which for the first time includes adolescents and redefines the right to health as a three-part quest: 1) to Survive, by ending preventable causes of death; 2) to Thrive, by ensuring health and well-being; and 3) to Transform, by expanding enabling environments in partnership with multiple sectors to sustainably improve inter-generational health. Examining both research priority exercises through this lens invites researchers to imagine adolescent health as a hierarchy of needs — from medical treatment, to community wellness, to health systems sustainability — and to frame their own questions accordingly. Current questions reflect strong interest in Survive and Thrive, with perhaps less developed recognition of the utility or relevance of the Transform objective [26].

### Limitations

This study has several limitations. First, as the first study of its kind, no guidelines exist on how best to conduct a mixed-methods synthesis of research priority-setting exercises based on the CHNRI methodology. However, we used well-established research methods such as content analysis [21], word cloud analysis [19], and meta-synthesis [22] as the basis of this mixed-methods synthesis. Second, the word cloud described the frequency of occurrence of words, but this does not necessarily reflect the importance or priority of these words, and word cloud methods cannot explore complex topics (such as a topic that requires several words to explain). Even so, the context and purposes of the two adolescent health research priority-setting exercises were the same, allowing word cloud analysis to be applied. Strengths of the word cloud include the presentation of the most common words using engaging visual depictions. Third, the two adolescent health research priority-setting exercises were generated by different groups of experts using distinct selection processes with slightly modified scoring criteria. For example, the adolescent sexual and reproductive health research exercise used equal weighting for the scoring criterion whereas the eight areas of adolescent health used weighting according to published guidelines from CHNRI stakeholders [15]. In addition, the exercises produced different numbers of priority research questions: the first exercise [3], produced 36 “top-ranked” research questions spread across seven domains of adolescent sexual and reproductive health including HIV and gender-based violence, and the second exercise [4] produced 80 research questions in eight other areas of adolescent health. Although the adolescent health research priority-setting exercise allowed more top-ranked questions in each domain (n = 10), the domains covered in the adolescent sexual and reproductive health CHNRI exercise were more narrowly focused (eg, integration of family planning and HIV-related services) than in the subsequent exercise that covered eight other health areas (eg, injuries and violence). Although the top questions from the two research priority-setting exercises were complementary, they were not originally designed to be merged. Therefore, in this synthesis, we did not quantitatively compare the scoring of the questions from the two different exercises and were not able to produce an overall numerical ranking of top questions across all areas of adolescent health research. However, the goal of the qualitative synthesis was to identify themes across health areas and disciplines related to adolescent health research, which we were able to accomplish using qualitative methodologies such as content analysis. Finally, although the CHNRI methodology has been used in over 50 research prioritization exercises, it should be noted that these exercises represent a diversity of topics, contexts, and purposes [8,10]. The two exercises synthesized in this study were both uniquely on the topic of adolescent health research, focused in the context of LMICs, and conducted by WHO to stimulate adolescent health research globally. It may not be appropriate to synthesize other CHNRI exercises that have differing topics, contexts, or purposes.

This study advances the CHNRI methodology by conducting the first mixed-methods synthesis of multiple research priority-setting exercises. The synthesis adds to the lessons learned from the two individual priority-setting exercises by identifying a focus on effectiveness research for interventions, the delivery platforms that the top-ranked questions related to, and the vulnerable sub-populations of adolescents that were prioritized for research. The framing of these adolescent research priorities within the larger United Nations Global Strategy Survive, Thrive and Transform framework can help researchers and funders further focus their efforts for adolescent health research in LMICs.
