# Supplementary material for: Research priorities for adolescent health in low- and middle-income countries: A mixed-methods synthesis of two separate exercises
Source: J Glob Health. 2018 Feb 17;8(1):010501. doi: 10.7189/jogh.08.010501 (PMC5825976; doi:10.7189/jogh.08.010501)
Supplement: Online Supplementary Document [file jogh-08-010501-s001.pdf]

Online Supplementary Document

Nagata et al. Research priorities for adolescent health in low- and middle-income countries: A mixed-methods synthesis of two separate exercises

J Glob Health 2018;8:010501

Table S1. Priority questions for adolescent health organized by platform

**School**
How best can school-based 'safe routes to school' initiatives be scaled up to include larger numbers of schools and to be incorporated with community-based initiatives?
Can swimming and water survival training be effectively implemented in late primary or early secondary school in LMICs in high risk/high need communities, and do such programmes have an intergenerational effect by also protecting subsequent generations of children by transfer of awareness, skills, knowledge, rescue techniques?
What interventions can be integrated into community settings (e.g. schools) to address gender- based violence and its related reproductive outcomes?
How can school-based and community-based programmes for STI counselling and testing, HPV vaccination and sex education be scaled up?
How can mental health promotion interventions in schools be scaled up in LMICs?
What is the prevalence of adolescent under-nutrition and over-nutrition by risk/protective factors such as sex, urban/rural residence, schooling, access to green spaces, access to food and socio-economic strata in different world regions?
What are the most effective interventions for preventing and reducing overweight/obesity in adolescents either in schools or out-of-school?
What is the best (feasibility, cost, acceptability, effectiveness, sustainability) design of a school-based intervention that aims to engage and gain the support of students, parents and teachers for young people to take the recommended 60 minutes of physical activity daily, and to ensure that there are at least two physical education (PE) classes within schools per week, with at least 50% of the time for PE classes spent in moderate-to-vigorous intensity physical activity)?
How best can the capacity of the education sector be improved to deliver high quality physical education programs within schools?
How does one best implement a sustainable, structured physical activity program for adolescents in schools and out of schools in LMICs?
How best can parents, teachers, and policymakers be engaged in creating physical activity-friendly school environments for children and adolescents?
What are the effects of daily physical education and recreation on total physical activity levels, physical fitness, cognitive development, and school performance among children and adolescents?
Are there distinct patterns of and factors leading to substance use (tobacco and other substances) among in- and out-of-school female adolescents and male adolescents? (These include: context of use, preferred substance, use related practices, among others)
What are the key interventions that should be part of routine school health service provision?
How do programmes that aim to keep girls in school longer through measures such as conditional cash transfers affect the prevalence of gender-based violence?
What is the impact of “healthy schools” initiatives on the reduction in gender-based violence?
Does the provision of comprehensive sex education at school: (i) reduce adolescent pregnancies, (ii) increase health-care seeking behaviour among adolescents, or (iii) reduce the incidence of STIs, including HIV infection?
**Primary care**
What are effective interventions to prevent and treat mental health problems of adolescents that can be delivered at primary care level in LMICs?
What are the costs and benefits of integrating management of child and adolescent mental disorders with other child and adolescent health care delivery platforms?

How can mental health and psychosocial support (including identification, support and basic management of relevant conditions) be integrated with adolescent friendly services, general health, reproductive health etc?

What are effective and evidence-based screening, prevention, and treatment interventions for adolescents with neurodevelopmental disorders and intellectual disabilities (and their families) that can be delivered in various settings?

Is it possible to develop a low-cost, accurate blood glucose point-of-care test (end-user cost USD 0.10 or less per test)?

What is the efficacy and effectiveness of a screening instrument linked to a brief intervention for alcohol use among adolescents for use in primary care settings?

How can primary health care services be designed to most effectively meet the unique health needs of adolescents?

What is the coverage of primary health care services for adolescents?

What is the effectiveness of different models of provision of primary care by community health workers in settings that are accessible and acceptable to adolescents?

What aspects of the delivery of HIV testing and counselling services are most important from the perspective of adolescents: the speed of the results; confidentiality and anonymity; the social and health services offered; the counselling offered; whether or not they are integrated into the health system?

What are the key barriers faced by adolescents to access TB and TB/HIV diagnostic and treatment services in high and low income countries, and how can these be overcome?

Do adolescent girls and adult women receive different antenatal, delivery and postnatal care? If so, how and why?

Community

What is the effectiveness of different models of provision of primary care by community health workers in settings that are accessible and acceptable to adolescents?

What are the true rates (based on empirical data not models) of mortality and DALYs lost from diarrhoeal disease and from lower respiratory tract infections in adolescents (10-14y and 15-19y) by sex, SES, rural/urban, by HIC/UMIC/LMIC/LIC, and by world region?

What are the risk and protective factors at various levels (individual, family, peer/social, community) for injuries and violence among adolescents LMICs?

How best can school-based 'safe routes to school' initiatives be scaled up to include larger numbers of schools and to be incorporated with community-based initiatives?

Can swimming and water survival training be effectively implemented in late primary or early secondary school in LMICs in high risk/high need communities, and do such programmes have an intergenerational effect by also protecting subsequent generations of children by transfer of awareness, skills, knowledge, rescue techniques?

How can community-based adolescent obesity prevention programmes be better implemented and scaled up?

What are adolescents' preferences for type of physical activities by community and the relationship of this to their cultural background?

What are the most effective strategies for evaluating community-based interventions for reducing the burden of substance use among adolescents?

What interventions can be integrated into community settings (e.g. schools) to address gender- based violence and its related reproductive outcomes?

How feasible, effective and sustainable is the training of community-based health workers on identification and referral of cases of gender-based violence?

How can school-based and community-based programmes for STI counselling and testing, HPV vaccination and sex education be scaled up?

Parenting

What are effective and evidence-based screening, prevention, and treatment interventions for adolescents with neurodevelopmental disorders and intellectual disabilities (and their families) that can be delivered in various settings?

What are the risk and protective factors at various levels (individual, family, peer/social, community) for injuries and violence among adolescents LMICs?

What specific behaviour modification strategies are effective in reducing risk taking behaviour and exposure to injury and violence?

What is the best (feasibility, cost, acceptability, effectiveness, sustainability) design of a school-based intervention that aims to engage and gain the support of students, parents and teachers for young people to take the recommended 60 minutes of physical activity daily, and to ensure that there are at least two physical education (PE) classes within schools per week, with at least 50% of the time for PE classes spent in moderate-to-vigorous intensity physical activity)?

How best can parents, teachers, and policymakers be engaged in creating physical activity-friendly school environments for children and adolescents?

What is the effectiveness of parenting programmes in the prevention of mental health disorders in adolescents?

What is the effectiveness of programmes assisting parents in the management of adolescents with substance use disorders?
**Internet, mobile phones, electronic, social media**
What are effective and evidence-based screening, prevention, and treatment interventions for adolescents with neurodevelopmental disorders and intellectual disabilities (and their families) that can be delivered in various settings?
What types of communication strategies work best to actually change the key behaviours that put adolescents at increased risk of injuries?
How can new technologies such as cell phones and the Internet be used effectively to provide information, referral and treatment for adolescents?
How can we develop health systems to interact with adolescents in both traditional (in person) and innovative (virtual) ways to promote positive health choices and prevent illness?
How do adolescents use information technologies (e.g. web, traditional and social media), and what implications does this have for their health behaviour, and for the design of interventions?
**Peer Education**
What is the potential contribution of peer-led interventions for improving retention in care among adolescents with TB and/or HIV?
What is the impact of peer education on reducing substance abuse in young people?

Questions in highlighted rows appear across multiple platforms

Table S2

**Appendix B.** Priority questions for adolescent health organized by Survive, Thrive, Transform framework from the Global Strategy for Women's, Children's, and Adolescents' Health						
**Survive**	**Thrive**	**Transform**	**Survive + Thrive**	**Survive + Transform**	**Thrive + Transform**	**Survive + Thrive + Transform**
What are the risk and protective factors at various levels (individual, family, peer/social, community) for injuries and violence among adolescents LMICs?	What types of communication strategies work best to actually change the key behaviours that put adolescents at increased risk of injuries?	How can new technologies such as cell phones and the Internet be used effectively to provide information, referral and treatment for adolescents?	What are effective and evidence-based screening, prevention, and treatment interventions for adolescents with neurodevelopmental disorders and intellectual disabilities (and their families) that can be delivered in various settings?	How best can school-based 'safe routes to school' initiatives be scaled up to include larger numbers of schools and to be incorporated with community-based initiatives?	What is the best (feasibility, cost, acceptability, effectiveness, sustainability) design of a school-based intervention that aims to engage and gain the support of students, parents and teachers for young people to take the recommended 60 minutes of physical activity daily, and to ensure that there are at least two physical education	How can primary health care services be designed to most effectively meet the unique health needs of adolescents?

					(PE) classes within schools per week, with at least 50% of the time for PE classes spent in moderate-to-vigorous intensity physical activity)?	
What specific behaviour modification strategies are effective in reducing risk taking behaviour and exposure to injury and violence?	To what extent do strategies that have been shown to reduce one form of violence (e.g., bullying) effectively prevent other forms of violence that youth experience (e.g., partner violence, sexual violence, suicidal behaviour)?	How can we develop health systems to interact with adolescents in both traditional (in person) and innovative (virtual) ways to promote positive health choices and prevent illness?	What is the effectiveness of parenting programmes in the prevention of mental health disorders in adolescents?	How do interventions devised for the management of NCDs in high income countries be used for adolescents in low- and middle-income countries translate globally?	How best can parents, teachers, and policymakers be engaged in creating physical activity-friendly school environments for children and adolescents?	What are effective interventions to prevent and treat mental health problems of adolescents that can be delivered at primary care level in LMICs?
Can swimming and water survival training be effectively implemented in late primary or early secondary school in	Do adolescent girls and adult women receive different antenatal, delivery and	How do adolescents use information technologies (e.g. web, traditional and	What are the rates of development of anti-tuberculosis drug resistance in adolescents?	How best can brief alcohol interventions be combined with brief	What are the effects of daily physical education and recreation on total physical	How can mental health and psychosocial support (including

LMICs in high risk/high need communities, and do such programmes have an intergenerational effect by also protecting subsequent generations of children by transfer of awareness, skills, knowledge, rescue techniques?	postnatal care? If so, how and why?	social media), and what implications does this have for their health behaviour, and for the design of interventions?		violence reduction interventions and be effectively delivered through the health system, when adolescents present with injuries?	activity levels, physical fitness, cognitive development, and school performance among children and adolescents?	identification, support and basic management of relevant conditions) be integrated with adolescent friendly services, general health, reproductive health etc?
What is the potential contribution of peer-led interventions for improving retention in care among adolescents with TB and/or HIV?	What is the effectiveness of programmes assisting parents in the management of adolescents with substance use disorders?	What interventions can be integrated into community settings (e.g. schools) to address gender- based violence and its related reproductive outcomes?	How effective and cost-effective is the integration of HIV and TB surveillance to enhance early detection and case management in adolescents?	What are the barriers and facilitators to increasing compliance with motorcycle helmet legislation?	What is the efficacy and effectiveness of a screening instrument linked to a brief intervention for alcohol use among adolescents for use in primary care settings?	
What are the key barriers faced by adolescents to access TB and TB/HIV diagnostic and treatment services in	What is the impact of peer education on reducing substance abuse in young people?	How can mental health promotion interventions in schools be	Can overall duration of TB treatment and/ or frequency of TB medication dosing be reduced to facilitate adherence and	What would be the most cost-effective, affordable and feasible	What are the most cost-effective interventions to decrease multiple	

high and low income countries, and how can these be overcome?		scaled up in LMICs?	improve rates of treatment completion among adolescents?	package of interventions for promotion of mental health and prevention of mental health disorders among adolescents?	health-risk behaviours and conditions and promote healthy behaviours?	
What aspects of the delivery of HIV testing and counselling services are most important from the perspective of adolescents: the speed of the results; confidentiality and anonymity; the social and health services offered; the counselling offered; whether or not they are integrated into the health system?	How can school-based and community-based programmes for STI counselling and testing, HPV vaccination and sex education be scaled up?	How best can the capacity of the education sector be improved to deliver high quality physical education programs within schools?	What are the risk and protective factors for mental health problems among adolescents with developmental disorders (e.g. developmental delay, autism) in LMIC?	What are the mortality and morbidity rates and their causes amongst adolescents with diabetes in low- and middle-income countries?		
What are treatment adherence rates, and what are the risk factors for non-adherence or default, among adolescents on long-term treatment for TB?	What is the prevalence of adolescent under-nutrition and over-nutrition by risk/protective factors such as sex, urban/rural	What platforms and strategies are most effective to reach and help the most vulnerable adolescents	What are the models of adolescent mental health services in LMICs that can be delivered to particularly vulnerable adolescents (refugees, out-of-school youth,			

	residence, schooling, access to green spaces, access to food and socio-economic strata in different world regions?	(eg. those not in school, slum dwellers and/or those in poor families)?	young people living with HIV, exposed to gender-based violence, youth in armed conflict)?			
What are the true rates (based on empirical data not models) of mortality and DALYs lost from diarrhoeal disease and from lower respiratory tract infections in adolescents (10-14y and 15-19y) by sex, SES, rural/urban, by HIC/UMIC/LMIC/LIC, and by world region?	What are the most effective interventions for preventing and reducing overweight/obesity in adolescents either in schools or out-of-school?	How do programmes that aim to keep girls in school longer through measures such as conditional cash transfers affect the prevalence of gender-based violence?	What interventions are effective in improving access to the medicines and supplies needed to manage diabetes and other endocrine disorders in adolescents?			
Is it possible to develop a low-cost, accurate blood glucose point-of-care test (end-user cost USD 0.10 or less per test)?	How does one best implement a sustainable, structured physical activity program for adolescents in schools and out of schools in LMICs?	What is the impact of "healthy schools" initiatives on the reduction in gender-based violence?	Are there biological changes (markers) heralding NCDs in adolescents, and how early can these be detected?			
What proportion of young women who test positive for HIV in antenatal or delivery care: (i) receive and take drugs for PMTCT;	What are the key interventions that should be part of routine school health service provision?	What are the costs and benefits of integrating management of child and				

(ii) are assessed to determine if they need lifelong HAART; (iii) are started on lifelong HAART if clinically indicated?		adolescent mental disorders with other child and adolescent health care delivery platforms?				
Which programmatic interventions developed to improve adolescent retention in care and treatment adherence for other communicable diseases (i.e. HIV) would be useful for application in TB programs?	Does the provision of comprehensive sex education at school: (i) reduce adolescent pregnancies, (ii) increase health-care seeking behaviour among adolescents, or (iii) reduce the incidence of STIs, including HIV infection?	How feasible, effective and sustainable is the training of community-based health workers on identification and referral of cases of gender-based violence?				
What is the incidence and burden of TB among younger (10-14y) and older (15-19y) adolescents in the world, by sex particularly among adolescents with HIV, and what proportion of the adolescents have drug resistant TB?	What is the effectiveness of different models of provision of primary care by community health workers in settings that are accessible and acceptable to adolescents?	What are the risk factors contributing to adolescents' substance use in the different world regions?				

What is the best way to help adolescents to adhere to TB medication when they are also taking ARVs?	What are the risk and protective factors associated with the increased risk of burn injuries among adolescent girls in many South Asian countries?	How does consumption of alcohol and other substances among adolescents change over time when alcohol and other drug policies change?				
What are effective interventions addressing self-harm/suicide in adolescent girls in LMICs?	How can community-based adolescent obesity prevention programmes be better implemented and scaled up?	What strategies might reduce gender-based violence among adolescent sex workers?				
Can interventions for the management of NCDs that have been shown to be effective in adults be used directly in adolescents?	What are the most effective strategies for evaluating community-based interventions for reducing the burden of substance use among adolescents?					
What proportion of children born with sickle cell disease	What interventions can be used to facilitate continuity of care					

survive into and through adolescence?	for mobile adolescent populations?					
What are existing effective programs in low- and middle-income countries that manage adolescents with diabetes, determining successful strategies?	What strategies can improve the use of antenatal care, skilled birth attendants, PMTCT and postnatal care by adolescents in resource-poor settings?					
How does the prevalence of non-communicable diseases change during adolescence by age and sex?	Do programmes that promote postnatal family planning for adolescent mothers reduce subsequent unwanted pregnancies in this group?					
What factors facilitate uptake, retention and adherence and minimize treatment failure among adolescents?	What is the coverage of primary health care services for adolescents?					
How do user fees affect access to, use of and retention in treatment among adolescents living with HIV?	What barriers do health-care providers face when trying to offer contraception services to					

	unmarried adolescents?					
What factors influence the disclosure of HIV status to others among adolescents?	What are the causes of anaemia among adolescent girls and how does this vary by region?					
	What are the relationships between early pregnancy and stunting, anaemia, and NCD risk (overweight, diabetes, hypertension)?					
	What social and behaviour change communication platforms are the most effective to reach adolescents to help them to improve their diet?					
	How does the burden of disease from nutritional causes for adolescent boys and girls vary by country and within countries, and by socio-economic status?					

	How do nutrition interventions during adolescent antenatal and postnatal visits impact on birth outcome, maternal, neonatal and child health?					
	What ante-natal interventions can be developed to help support the specific health and nutritional needs of adolescent pregnant girls in developing countries?					
	How do we improve compliance and acceptability of iron supplementation programmes among adolescents (eg. design supplements with lower dose iron, different form such as powders, fewer side effects etc)?					

	What are scalable and sustainable approaches to improve physical activity in children and adolescents, particularly in low-to-middle income countries?					
	What prevention and treatment services related to substance use are acceptable to adolescents?					
	What is the effectiveness of implementation of youth friendly services interventions on substance use?					
	What factors (including barriers and facilitators) are associated with the utilization of maternal health services (antenatal, intrapartum, postpartum) and neonatal care by adolescents in different settings?					

	What pregnancy outcomes (maternal and neonatal) among adolescents are related to mode of delivery, presence of a skilled birth attendant at delivery and care of infants up to 6 months of age?					
	What strategies can delay first births among married adolescents?					
	Through what mechanisms can the provision of regular and emergency contraceptives to adolescents be financed or subsidized?					
	What strategies can increase consistent and effective condom use among both male and female adolescents?					

	In settings with high rates of pregnancy in adolescence, what factors protect adolescents from unwanted and/or unsafe pregnancy?					
	How does the provision of contraceptive methods (especially long-acting, reversible methods) as part of post-abortion care affect unintended pregnancy and repeat abortion rates among adolescents?					
	What interventions are effective for informing adolescents about the availability and safe use of misoprostol?					
	How does cost influence adolescents' abortion-seeking behaviour?					

	How much awareness of abortion law, access to safe abortion services and post-abortion care exists among adolescents?					
	What do adolescents know about less invasive procedures for pregnancy termination and post- abortion care (e.g. misoprostol), and to what extent do they have access to them or use them?					
	What modalities for delivering integrated HIV/FP services to adolescent boys work best?					
	What are the most effective and affordable models for delivering integrated contraception and HIV services and information to					

	young married couples?					
	What female-controlled methods for preventing both STIs and pregnancy can be developed and tested?					
	How much do young female sex workers and injecting drug users need and use contraceptives??					
	What alternative dosing schedules can facilitate HPV vaccine delivery in low-resource settings?					
	What are the most effective, efficient and sustainable ways to deliver vaccination against HPV?					
	How can adolescents who do not use available STI services (e.g. conditional cash					

	transfers, mobile clinics) be reached?					
	What is the cost-effectiveness of HIV/STI screening programmes among adolescents at highest risk?					
	How can the incorporation of syphilis testing in SRH and maternal health services be optimized to ensure that all adolescents, including pregnant girls, get screened and treated?					
